# A case report of recurrent endometrial cancer invading the rectum and bladder with concurrent primary rectal cancer treated with total neoadjuvant therapy and pelvic exenteration

**DOI:** 10.1093/jscr/rjaf529

**Published:** 2025-07-17

**Authors:** Batoul Mazraani, Quinn Simpson, Marine Bolliet, Scarlett Hao, Harry Wasvary

**Affiliations:** Oakland University William Beaumont School of Medicine, 586 Pioneer Dr, Rochester, MI 48309, United States; Oakland University William Beaumont School of Medicine, 586 Pioneer Dr, Rochester, MI 48309, United States; Department of Surgery, Henry Ford Providence Hospital-Michigan State University College of Human Medicine, 16001 W Nine Mile Rd, Southfield, MI 48075, United States; Department of Colorectal Surgery, Corewell Health William Beaumont University Hospital, 3601 W 13 Mile Rd, Royal Oak, MI 48073, United States; Department of Colorectal Surgery, Corewell Health William Beaumont University Hospital, 3601 W 13 Mile Rd, Royal Oak, MI 48073, United States

**Keywords:** endometrial cancer, bladder cancer, rectal cancer, cystectomy, uretero-sigmoid conduit, recurrence

## Abstract

Endometrial cancer, the fourth most common cancer affecting women in the USA, typically recurs locally or can invade regional lymph nodes and the peritoneum. A 66-year-old female with history of Stage 1b endometrial adenocarcinoma was found to have rectal adenocarcinoma on surveillance colonoscopy. Cross-sectional imaging demonstrated a vaginal cuff mass with bladder wall thickening and unilateral hydronephrosis. A bladder mass was identified and partially resected on cystoscopy; pathology was indicative of urothelial carcinoma. The final surgical specimen demonstrated endometrial cancer as the primary culprit in all three organs, confirmed by additional immunohistochemical staining of the previous bladder resection specimen. The rarity of hollow organ involvement in locoregional endometrial cancer recurrence can engender misdiagnosis, potentially leading to discordant treatment and suboptimal outcomes. A high index of suspicion in conjunction with multidisciplinary discussion may have prompted additional immunohistochemical testing to obtain the correct diagnosis earlier in the patient’s clinical course.

## Introduction

Endometrial carcinoma is the most common gynecologic malignancy in developed countries. In patients with endometrial cancer presenting with multiple areas of pelvic tumor burden, it is necessary to determine if the malignancy is metastatic from the primary or if the tumors represent multiple synchronous primary neoplasms. Radiographic and endoscopic evaluation alone cannot differentiate, rather a combination of various tumor characteristics, imaging, and tissue pathology must be considered in concert to reduce misdiagnosis [1]. Endometrial carcinoma primarily recurs at the vagina or within the peritoneum and intraabdominal lymph nodes [2]. Direct local spread can occur to nearby structures including the rectum and the bladder but this has only been described in a handful of case reports [3–5].

While it can be challenging to diagnose and differentiate recurrent endometrial carcinoma from synchronous primary tumors, immunohistochemical (IHC) staining is a tool that can help establish a diagnosis. This is the first case in literature to describe endometrial cancer recurrence in the vaginal cuff with concurrent rectal and bladder involvement in addition to a synchronous rectal tumor. The initial diagnosis of triple primary tumors was found to be incorrect on final pathology with the bladder invasion of recurrent endometrial carcinoma masquerading as urothelial cancer. This case highlights the critical importance of pretest suspicion and careful selection of IHC staining to obtain correct diagnoses.

## Case report

The patient was a 66-year-old female with a past medical history of anxiety, type 2 diabetes, obesity (BMI 31), and hypertension. She had been diagnosed with Stage I endometrial carcinoma 6 years prior. Baseline cancer antigen 125 (Ca-125) was 11 U/ml. Family history included colon cancer in her mother diagnosed at age 69. She underwent total laparoscopic hysterectomy with bilateral salpingo-oophorectomy and pelvic lymph node assessment and excision. Pathology revealed Grade 1 tumor with negative margins and 0/23 lymph nodes involved. She received adjuvant vaginal cuff brachytherapy. She developed abdominal pain: imaging revealed abdominal implants which were biopsy-proven recurrence. Due to the COVID-19 pandemic, she began with three cycles of carboplatin/paclitaxel, followed by cytoreductive surgery, and three more chemotherapy cycles.

Follow-up imaging demonstrated persistent disease at the vaginal cuff, a raised intravesicular lesion on cystoscopy, and negative proctoscopy. She was started on letrozole/everolimus, with partial response.

**Figure 1:**
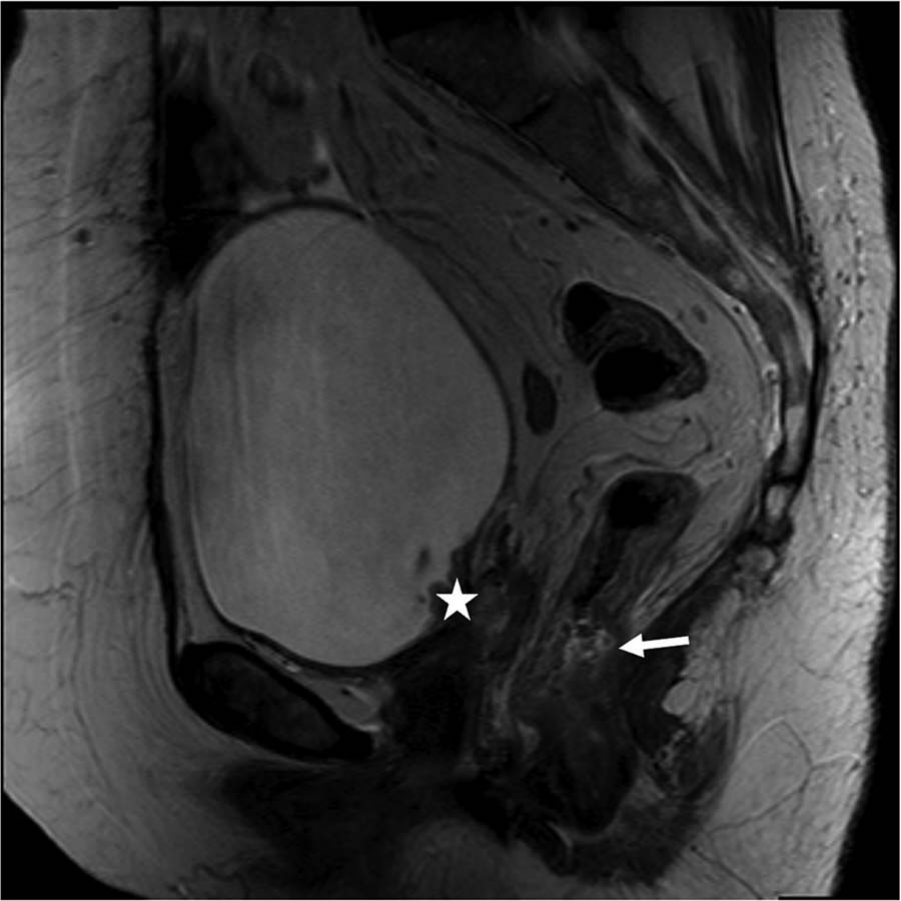
Rectal MRI, sagittal view. Star marks vaginal cuff mass with bladder invasion; arrow marks rectal tumor.

At her next colonoscopy, a palpable posterior rectal mass was biopsied confirming rectal adenocarcinoma with positive caudal type homeobox 2 (CDX-2) and negative paired box gene 8 (PAX-8). The tumor was mismatch repair (MMR) proficient and microsatellite stable. Imaging showed progression of the vaginal cuff mass extending to the bladder with right hydronephrosis. Magnetic resonance imaging (MRI) indicated a cT3 cN0 rectal tumor separate from the vaginal recurrence (Fig. 1). Cystoscopy revealed a tumor in the right hemitrigone, biopsy confirmed urothelial carcinoma.

She began total neoadjuvant therapy (TNT) with FOLFOX and short-course radiotherapy, with planned pelvic exenteration.

**Figure 2:**
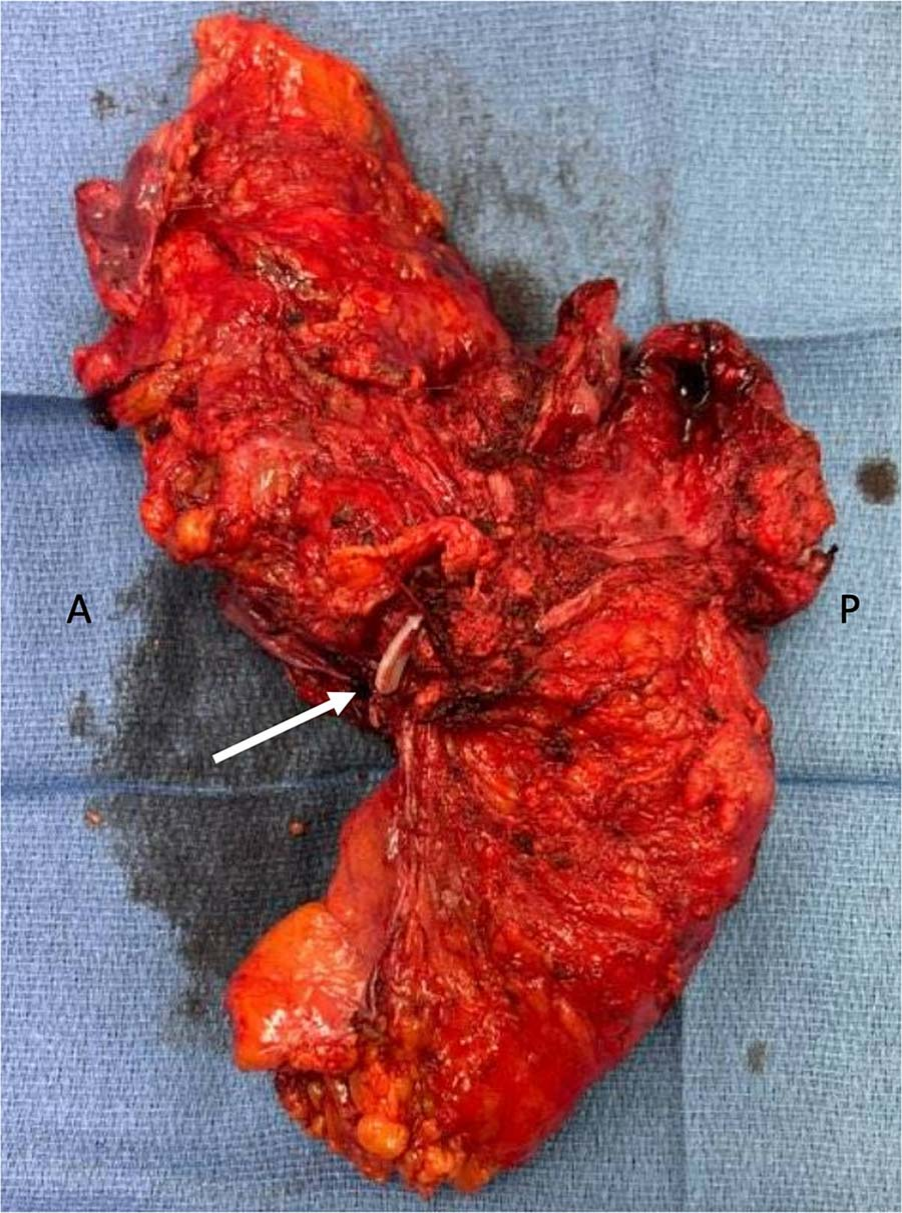
En-bloc resected specimen, right lateral view. Arrow indicates hemolok clip placed on ureter; letters A and P indicate anterior and posterior, respectively.

Genetic testing revealed a variant of unknown significance of the BARD1 gene. Post-treatment MRI showed yMR-T2 tumor. Sigmoidoscopy revealed a rectal ulcer, and she underwent robotic abdominoperineal resection (APR), cystectomy, and vaginectomy (Fig. 2). A uretero-sigmoid conduit and end colostomy were created. Final pathology revealed high-grade endometrial adenocarcinoma involving bladder wall, rectum, anus, vagina/cuff, and perirectal tissues. Negative for urothelial carcinoma, the original bladder biopsy was reexamined: staining GATA binding protein 3 (GATA-3) negative, PAX-8 positive, more consistent with endometrial origin. No rectal primary was identified, but two lymph nodes contained malignant cells with colorectal IHC features.

Four months later with worsening ascites, pleural effusions, and abdominal wall implants, she was found to have recurrent Mullerian adenocarcinoma. She started palliative chemotherapy, but transitioned to hospice, ultimately expiring.

## Discussion

Direct endometrial adenocarcinoma invasion often occurs through the rectovaginal septum. In the present case, since the rectal primary was identified radiographically on the posterior wall as a separate process from the vaginal cuff, it is likely this represented a synchronous primary rectal cancer. This was further confirmed by the colorectal IHC staining pattern of the two involved lymph nodes. With complete clinical and pathologic response rates seen in Stage II/III rectal cancers treated with TNT [6], TNT remains a feasible treatment for primary rectal cancer in the setting of recurrent endometrial carcinoma. She was not eligible for watch-and-wait due to persistent endoscopic ulceration and therefore underwent an APR [7].

Secondary bladder primaries post-radiation for endometrial carcinoma have been described [8]. In cases of true multiple synchronous primary cancers, genetic testing is paramount. While mutations of the BARD1 gene are classically associated with breast cancer, there are variants that may predispose patients to malignancy given the presumptive role in DNA repair [9].

IHC can help distinguish endometrial from primary colonic and urologic tumors, however deciding to stain for certain markers requires pretest suspicion [1]. Primary colon cancer is usually cytokeratin-7 negative and cytokeratin-20 positive, whereas endometrial cancers are usually cytokeratin-7 positive and cytokeratin-20 negative, and urothelial cancers are often positive for GATA-3 (Table 1) [10]. In the presented case, initial pathology from biopsies were suspicious of simultaneous rectal, bladder, and vaginal cuff cancers. However, in our pathology report from the bladder tumor, GATA-3 was negative, consistent with endometrial origin. These additional stains were reexamined on the prior bladder biopsy and confirmed. Earlier IHC may have allowed correct preoperative diagnosis of endometrial invasion rather than a urothelial primary, although the impact on her outcome remains uncertain.

**Table 1:** Common IHC stains for colorectal, Mullerian, and urothelial origin tumors

	Colorectal	Mullerian	Urothelial
Cytokeratin-7	−	+	−
Cytokeratin-20	+	−	−
PAX-8	−	+	−
GATA-3	−	−	+
CDX-2	+	−	−
SATB-2	+	−	−

This is the first reported case of endometrial cancer recurrence at the vaginal cuff with concurrent bladder and rectal involvement, alongside a likely synchronous rectal cancer. The initial diagnosis of triple primary tumors was found to be incorrect on final pathology with the bladder invasion of recurrent endometrial carcinoma masquerading as urothelial cancer. Pretest suspicion is required for careful selection of IHC staining to obtain correct preoperative diagnoses.

### Lessons learned

A case of recurrent endometrial cancer with hollow organ invasion was treated with pelvic exenteration after neoadjuvant therapy for a synchronous rectal primary cancer and presumptive synchronous urothelial carcinoma. Final pathology revealed two malignancies: recurrent endometrial carcinoma and likely synchronous rectal cancer. Earlier IHC may have diagnosed an endometrial bladder metastasis, rather than a primary lesion. This may have avoided radical cystectomy and urinary diversion, thought the impact on her postoperative mortality is unknown.

## References

[1] Bochtler T, Löffler H, Krämer A. Diagnosis and management of metastatic neoplasms with unknown primary. Semin Diagn Pathol 2018;35:199–206. 10.1053/j.semdp.2017.11.013 29203116

[2] Kurra V, Krajewski KM, Jagannathan J, et al. Typical and atypical metastatic sites of recurrent endometrial carcinoma. Cancer Imaging 2013;13:113–22. 10.1102/1470-7330.2013.0011 23545091 PMC3613792

[3] Hubers JA, Soni A. A rare case of endometrial cancer metastatic to the sigmoid colon and small bowel. Case Rep Gastrointest Med 2017;2017:1–3. 10.1155/2017/9382486 PMC5676375 29209543

[4] Li M, Zheng W. Metastasis of endometrial adenocarcinoma masquerading as a primary rectal cancer: a rare case report with literature review. Medicine (Baltimore) 2023;102:e36170. 10.1097/MD.0000000000036170 37986305 PMC10659593

[5] Khadraoui W, Tymon-Rosario J, Nagarkatti N, et al. Robotic low anterior resection and partial bladder resection for management of locoregional endometrial cancer recurrence. J Minim Invasive Gynecol 2021;28:176–7. 10.1016/j.jmig.2020.06.006 32544562

[6] Goffredo P, Quezada-Diaz FF, Garcia-Aguilar J, et al. Non-operative management of patients with rectal cancer: lessons learnt from the OPRA trial. Cancers (Basel) 2022;14:3204. 10.3390/cancers14133204 PMC9264788 35804975

[7] Scott AJ, Kennedy EB, Berlin J, et al. Management of locally advanced rectal cancer: ASCO guideline. J Clin Oncol 2024;42:3355–75. 10.1200/JCO.24.01160 39116386

[8] Wen L, Zhong G, Ren M. Increased risk of secondary bladder cancer after radiation therapy for endometrial cancer. Sci Rep 2022;12:1032. 10.1038/s41598-022-05126-w 35058550 PMC8776857

[9] Adamovich AI, Banerjee T, Wingo M, et al. Functional analysis of BARD1 missense variants in homology-directed repair and damage sensitivity. PLoS Genet 2019;15:e1008049. 10.1371/journal.pgen.1008049 30925164 PMC6457558

[10] Schwartz LE, Khani F, Bishop JA, et al. Carcinoma of the uterine cervix involving the genitourinary tract: a potential diagnostic dilemma. Am J Surg Pathol 2016;40:27–35. 10.1097/PAS.0000000000000524 26426382

